# Description and prediction of the development of metabolic syndrome in Dongying City: a longitudinal analysis using the Markov model

**DOI:** 10.1186/1471-2458-14-1033

**Published:** 2014-10-04

**Authors:** Xiaoxiao Chen, Qicai Chen, Lili Chen, Pengpeng Zhang, Juan Xiao, Shumei Wang

**Affiliations:** Department of Epidemiology and Biostatistics, School of Public Health, Shandong University, Jinan, China; Department of Prevention and Health Care, Dongying Shengli Oilfield Central Hospital, Dongying, China; Department of Nutrition and Food Safety, Zhejiang Center for Disease Control and Prevention, Hangzhou, China; Tianjin Entry-Exit Inspection and Quarantine Bureau, Tianjin, China

**Keywords:** Metabolic syndrome, Markov model, Dongying City

## Abstract

**Background:**

Metabolic Syndrome (MS) is increasingly becoming a major worldwide clinical and public health issue. Thus it is extremely important to study the history of MS and search for the most likely component contributing to start the cascade of confusions of MS.

**Methods:**

A longitudinal cohort was involved which included the data of 7510 individuals who had at least two routine health check-ups in a six-year follow-up. Based on the data, a Markov model with each chain containing seven states (no component state, four isolated states, 2-component state, and MS state) was built. Annual transition probability was the mean of five probabilities for the transition between the given states between each pair of consecutive years.

**Results:**

The transition probabilities from the no component state to MS were higher in men than that in women in four age groups. In the young people (men <60 years and women <50 years), the probabilities to the overweight or obesity state and dyslipidemia state were the first two biggest probabilities in transition from no component to the rest six states. However, in the elderly population, the probabilities to hypertension state and 2-component state increased, even surpassed the above two states. The individuals initiating with 2-component states and the isolated hyperglycemia state were more likely to develop MS than the others.

**Conclusions:**

The Markov model was able to give a better description of the evolutionary history of MS, and to predict the future course based on past evidence. The occurrence of the MS process mostly began with overweight or obesity and dyslipidemia in young people. In the elderly population, many individuals initiating with hypertension or 2 components besides the above two states. Individuals with the isolated hyperglycemia had greater chances to develop MS than other isolated MS’ components.

**Electronic supplementary material:**

The online version of this article (doi:10.1186/1471-2458-14-1033) contains supplementary material, which is available to authorized users.

## Background

Metabolic syndrome (MS) is a clustering of metabolic risk factors, including obesity, hypertension, dyslipidemia, and insulin resistance [1, 2]. MS can raise the risk of cardiovascular disease (CVD) which is the leading killer in China [3–5]. Evidence also suggests that MS is strongly correlated with polycystic ovarian disease, nonalcoholic fatty liver disease, and some cancers [6–8]. In fact, MS is increasingly becoming a major worldwide clinical and public health issue [9–14].

Due to the great harm caused by MS, vast studies have been done on it. Previous studies mainly focused on searching for early MS’ biomarkers and risk factors, such as white blood cell count (WBC), serum uric acid (UA), gamma-glutamyl transpeptidase (GGT), alanine aminotransferase (ALT), physical inactivity, alcohol intake, and smoking habits [15–20]. Only a few studies focused on the onset process of MS [21, 22]. MS is defined as the existence of at least three of the four components at the same time, thus there are 16 different states and 256 transitions between states. Therefore, stating the history of MS and searching for the most likely component contributing to start the cascade of confusions are preconditions for preventing its development. Haring et al. used a network-based approach to show the prevalence and progression of MS components and their changes [22], but these predictions faced many barriers. The Markov model which is frequently used to represent a random process changing with time is an admitted method to simulate the natural history of chronic diseases [23]. Lee-Ching Hwang et al. applied a Markov model approach to predict the development of MS [21]. However, this study was only with young people and without intermediate process conditions, it was limited. Therefore, we conducted a Markov model with a six-year follow-up health check-up including different genders and age groups to describe the natural history of MS, determine gender and age differences in the natural progression of MS components and to predict the effect of different initial states on the development of MS.

## Methods

### Study samples

The participants for this study were collected during the period from September 2006 to September 2011 in the Health Management Center of Shengli Oilfield Central Hospital in Dongying City, located in eastern China. 7510 subjects who had at least two health check-ups in the six-year follow-up were involved in this study, the maximum follow-up was 6 years and the mean follow-up was 3.74 years. The Data for the first year enrolled in the study following was considered “baseline” no matter if that year was 2007, 2008, 2009 or 2010. Individual who had a history of coronary heart diseases, type I diabetes, familial hyperlipidemia, and those who did not provide complete information were excluded from the analysis.

The occurrence of chronic diseases such as hypertension and hyperglycemia is closely related to age and gender, so groups were classified by gender and age. Individuals were divided into four age groups, the 18–40 year group, the 40–49 year group, the 50–59 year group and the ≥60 year group, in two genders.

### Measurements

All subjects underwent a doctor’s interview, anthropometric and laboratory test. The doctor’s interview included age, family history, and medical history. The family history included certain genetic diseases such as familial hyperlipidemia and the medical history contained individuals’ medications such as taking antihypertensive drugs. The anthropometric variables contained weight, height and blood pressure. Weight and height were measured with the subjects wearing light clothes and no shoes. Body mass index (BMI) was obtained by dividing weight (kg) by squared height (m). Blood pressure was measured twice in a comfortable quiet sitting position with their right arm supported at the level of the heart by a calibrated mercury sphygmomanometer after at least 5 minutes rest. Laboratory tests contained fasting blood-glucose (FPG), triglyceride (TG), and high density lipoprotein cholesterol (HDL). FPG was tested with the glucose oxidase method and TG and HDL were measured by the enzymatic calorimetric test on blood samples taken from the antecubital vein from the individuals who were under at least 12 hours fasting conditions.

### Definition of metabolic syndrome

The criteria given by Chinese Medical Association Diabetes Branch (CDS) was adopted to define MS in our study [24]. Participants were diagnosed with MS if they had at least three of the following four risk factors: (1) overweight or obesity, BMI ≥25.0 kg/m^2^, (2) hypertension, systolic blood pressure ≥140 mmHg, or diastolic blood pressure ≥ 90 mmHg or previous diagnosed as hypertension, (3) dyslipidemia, fasting triglyceride (TG) ≥ 1.7 mmol/L(110 mg/dl), or fasting high-density lipoprotein cholesterol (HDL) < 0.9 mmol/L(35 mg/dl), (4) hyperglycemia, fasting blood-glucose (FPG) ≥ 6.1 mmol/L(110 mg/dl), or 2 h Post-meal glucose (PG) ≥ 7.8 mmol/L(140 mg/dl), or previously diagnosed as hyperglycemia.

### Markov model

**Figure 1:**
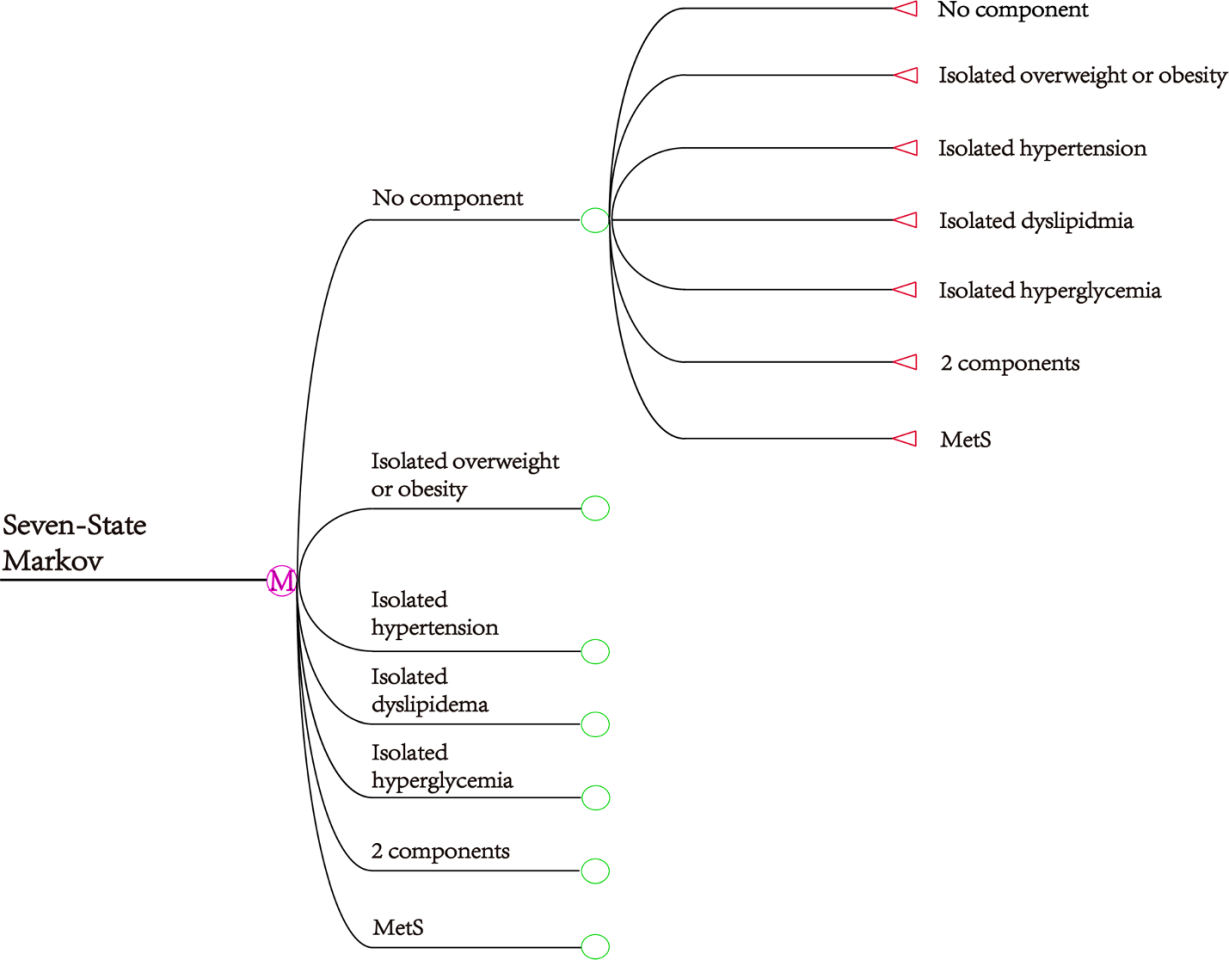
**A 7-state Markov model to describe the progression of metabolic components.**

7 states were included in each Markov chain: the no component state, isolated overweight or obesity state, isolated hypertension state, isolated dyslipidemia state, isolated hyperglycemia state, 2-component state, and MS state. Any two combinations of overweight or obesity, hypertension, dyslipidemia or hyperglycemia formed a 2-component state. These states were mutually exclusive and collectively exhaustive. A cycle-based Markov model was built and the graphical expression of the model was shown in Figure 1. The development of MS is a complex process and any state can develop into MS. Mutual transitions were allowed between the 7 states, so a reversible multistate Markov model was adopted. In every Markov cycle, the state could maintain or transition into any of the other 6 states.

Individuals in every age and gender group were assigned a probability of transitioning from one state to another or maintaining their states in each Markov cycle and these probabilities were calculated for annualized transitional probabilities. First, individuals were designed to have no component at the start of the model simulation in our study and they could change their states every year. Other Markov chains of different initial states, such as starting with the isolated overweight or obesity state, isolated hypertension state, isolated dyslipidemia state, isolated hyperglycemia state or 2-component state were then conducted.

We used the annualized transitional probabilities calculated from our six-year health check-up data to predict the effect of different initial states on the development of MS in the future 10 years in every age and gender group. A series of assumptions was necessary to simplify the process. (1) The future development depended only on an individual’s current state and had no relationship with any memory of prior states. (the Markov Chain assumption) (2) Each baseline transition rate was a constant, independent of when baseline occurred during the follow-up period.

### Statistical analysis

Means and percentages were calculated in the descriptive analysis for both baseline and follow-up and analysis of variance (ANOVA) and the Chi-square test were used to find differences for the continuous and dichotomous variables, respectively. Annual incidence rates were first separately calculated between each pair of consecutive years. Transition probabilities were the average of five annual incidence rates in every age and gender group which would increase the sample size and guarantee the stability of the results. All these statistical analysis were performed by SAS 9.1 and those two-sided p < 0.05 were considered statistically significant. TreeAge pro 2011 software was used to construct the Markov models and R2.14.2 was applied to draw figures.

### Ethic statements

This study was approved by the Ethics Committee of School of Public Health, Shandong University and informed oral consent was obtained from each participant. The ethical review boards approved because many individuals involved in this study were workers from rural areas, most of whom were illiterate. The consent processes were documented in recorder pens.

## Results

The general information of the baseline and each follow-up year was shown in Table 1. During the six-year follow-up, 7510 individuals were included in this study and the total follow-up years were 28,059 person-years with a mean 3.74 follow-up years per subject. MS prevalence increased with greater time since baseline. Additional file 1: Table S1 displayed the basic characteristics of the 7510 study individuals stratified by gender and age. All variables were associated with age.

**Table 1:** **General information of the study cohort**

Variables ^a^	Baseline (N=7510)	1-year follow-up (N=6480)	2-year follow-up (N=5420)	3-year follow-up (N=4644)	4-year follow-up (N=2899)	5-year follow-up (N=1106)	***P*** ^b^
Age (years)	41.02±10.26	41.86±10.21	43.57±10.16	45.38±10.16	47.67±10.86	46.53±7.72	<0.0001
Sex (male)	5118 (68.17%)	4299 (66.34%)	3592 (66.27%)	3041 (65.48%)	1879 (64.82%)	811 (73.33%)	<0.0001
Overweight or obesity	3088 (41.12%)	2642 (40.77%)	2346 (43.28%)	2057 (44.29%)	1198 (41.32%)	557 (50.36%)	<0.0001
Hypertension	2237 (29.79%)	2505 (38.66%)	2335 (43.08%)	2251 (48.47%)	1568 (54.09%)	672 (60.76%)	<0.0001
Dyslipidemia	2008 (26.74%)	1887 (29.12%)	1673 (30.87%)	1447 (31.16%)	954 (32.91%)	362 (32.73%)	<0.0001
Hyperglycemia	614 (8.18%)	729 (11.26%)	770 (14.21%)	772 (16.62%)	615 (21.21%)	237 (21.43%)	<0.0001
MS	893 (11.89%)	990 (15.28%)	985 (18.17%)	940 (20.24%)	661 (22.80%)	289 (26.13%)	<0.0001

^a^Values for continuous characteristics were expressed as mean ± SD; values for categorical data were expressed as n (%).

^b^P for each row testing the null hypothesis that values for six years were equal.

### The Markov model and transition probabilities

Each annual transition probability was the mean of five probabilities for the transition between the given states between each pair of consecutive years. The annual transition probabilities for the Markov chain model which displayed the natural history of MS divided by gender and age were presented in Tables 2, 3, 4, 5 and 6 and Additional file 1: Tables S2-S4. The transition probabilities from no component state to MS were higher in men than in women in four age groups and the same trend could be seen from MS state to MS. However, the gap between men and women gradually decreased with age. The probabilities for transition from isolated overweight state to MS for men were higher than women in the first two age groups, but an opposite result was demonstrated after age 50. The probabilities for transition from any isolated state to the 2-component state were always very high in the result that revealed the fact that MS components always simultaneously occurred. In men under 60 years old and women under 50 years old, the probabilities to the overweight or obesity state and dyslipidemia state were the first two biggest probabilities in transition from no component to the rest six states. However, in the elderly population, the probabilities to hypertension state and 2-component state increased, even surpassed the above two states.

**Table 2:** **Annual transition probabilities (%) in Markov chain models for men in the 18–40 year group**

Starting state	State after transition						
	No component	Isolated overweight or obesity	Isolated hypertension	Isolated dyslipidemia	Isolated hyperglycemia	2 components	MS
No component	72.07	9.48	4.55	7.68	0.58	4.55	1.09
Isolated overweight or obesity	13.51	52.25	1.03	3.35	0.51	24.07	5.28
Isolated hypertension	0	0	61.21	0	0	26.67	12.12
Isolated dyslipidemia	23.21	3.27	1.79	42.86	0.30	22.32	6.25
Isolated hyperglycemia	0	0	0	0	48.57	31.43	20.00
2 components	3.33	9.07	9.44	2.50	0.83	54.17	20.65
MS	0	0	3.78	0	0	23.92	72.30

**Table 3:** **Annual transition probabilities (%) in Markov chain models for women in the 18–40 year group**

Starting state	State after transition						
	No component	Isolated overweight or obesity	Isolated hypertension	Isolated dyslipidemia	Isolated hyperglycemia	2 components	MS
No component	86.38	3.71	2.15	3.76	0.80	2.30	0.90
Isolated overweight or obesity	37.81	44.27	0.50	4.98	0.50	9.45	2.49
Isolated hypertension	0	0	83.82	0	0	13.24	2.94
Isolated dyslipidemia	47.57	5.83	2.91	25.24	0.97	10.68	6.80
Isolated hyperglycemia	0	0	0	0	88.64	9.09	2.27
2 components	7.69	6.51	20.71	2.37	3.55	46.74	12.43
MS	0	0	25.00	0	0	42.31	32.69

**Table 4:** **Annual transition probabilities (%) in Markov chain models for men in the 40–49 year group**

Starting state	State after transition						
	No component	Isolated overweight or obesity	Isolated hypertension	Isolated dyslipidemia	Isolated hyperglycemia	2 components	MS
No component	68.15	8.30	4.26	10.54	1.57	5.72	1.46
Isolated overweight or obesity	13.17	52.23	0	3.13	0.22	25.45	5.80
Isolated hypertension	0	0	64.71	0	0	27.01	8.29
Isolated dyslipidemia	24.82	4.61	3.19	37.59	1.06	23.05	5.67
Isolated hyperglycemia	0	0	0	0	56.90	31.03	12.07
2 components	1.28	4.81	8.49	3.13	1.44	57.69	23.16
MS	0	0	2.60	0	0.21	22.56	74.64

**Table 5:** **Annual transition probabilities (%) in Markov chain models for women in the 40–49 year group**

Starting state	State after transition						
	No component	Isolated overweight or obesity	Isolated hypertension	Isolated dyslipidemia	Isolated hyperglycemia	2 components	MS
No component	82.05	5.45	3.70	4.61	0.84	2.65	0.70
Isolated overweight or obesity	27.50	54.16	1.25	4.17	0.42	11.25	1.25
Isolated hypertension	0	0	86.80	0	0	11.46	1.74
Isolated dyslipidemia	38.25	3.48	3.48	42.61	0	9.57	2.61
Isolated hyperglycemia	0	0	0	0	73.81	19.05	7.14
2 components	3.16	3.95	21.34	3.16	3.95	49.02	15.42
MS	0	0	4.88	0	2.44	36.59	56.09

**Table 6:** **Annual transition probabilities (%) in Markov chain models for men in the 50–59 year group**

Starting state	State after transition						
	No component	Isolated overweight or obesity	Isolated hypertension	Isolated dyslipidemia	Isolated hyperglycemia	2 components	MS
No component	62.71	9.90	5.94	7.92	3.96	7.59	1.98
Isolated overweight or obesity	18.62	46.28	2.66	4.79	0.53	20.21	6.91
Isolated hypertension	0	0	66.40	0	0	29.25	4.35
Isolated dyslipidemia	22.97	1.35	1.35	36.49	2.70	29.73	5.41
Isolated hyperglycemia	0	0	0	0	53.57	30.36	16.07
2 components	0.53	2.79	7.85	1.60	1.33	60.24	25.66
MS	0	0	2.13	0	0.15	21.46	76.26

#### Beginning with no component, any isolated component state, or 2-component state

**Figure 2:**
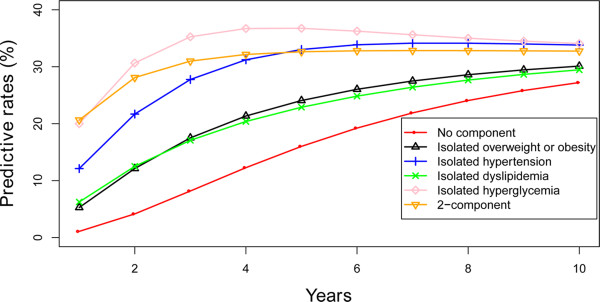
**The predictive development of MS according to various starting components in men in the 18–40 year group.**

**Figure 3:**
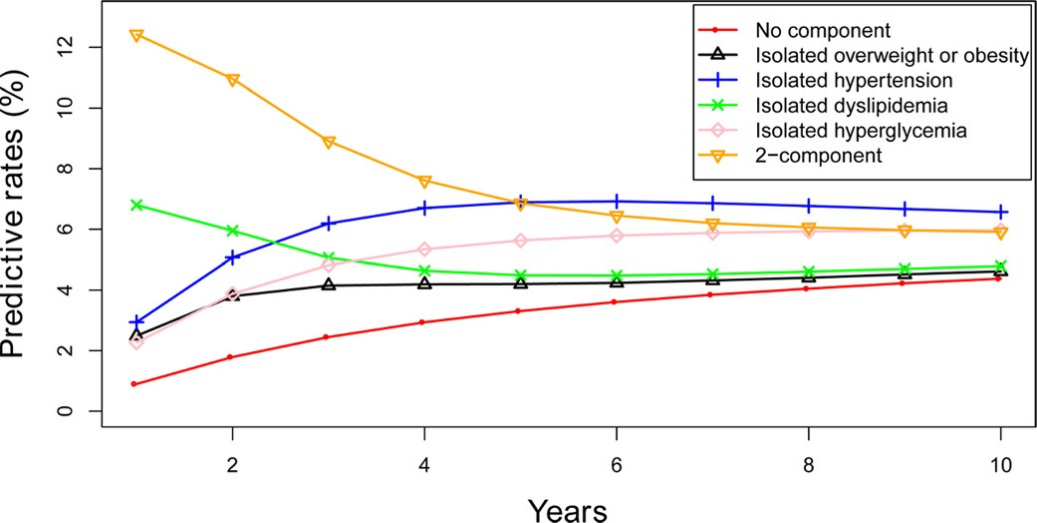
**The predictive development of MS according to various starting components in women in the 18–40 year group.**

The prediction of the development of MS with the hypothesis that individuals began with no component, any isolated component or 2-component state was shown in Figures 2 and 3 and Additional file 2: Figures S1-S6 divided by gender and age. Individuals beginning with any isolated component state or the 2-component state were more likely to develop MS than the individuals starting with no component in the prediction of 10 years. Similarly, there was a greater chance for the individuals starting with 2-component state and the isolated hyperglycemia state to develop MS than the others. Regardless of the beginning state, the possibility of developing MS after 10 years was extraordinarily similar.

#### Validation of the model

To validate the reality of our model, we compared the estimated prevalence of MS in the model with the empiric data after 5 years in each gender and age group. The outcomes were extremely similar.

## Discussion

As well-known methods for simulating the history of chronic diseases, Markov models were used in many studies. Feldman et al. used a longitudinal multistate Markov model to study the causal relationship between allergic sensitization and rhinovirus wheezing in 285 children at high risk for allergic disease and asthma [25]. Similarly, a Markov model analysis was used on a population-based cohort to depict clinical course and costs of care for Crohn's disease [26]. The patients are divided into different disease states and transition probability between any two states is given in the Markov model which applies to medical areas [27]. Seven states were included in our study and mutual conversion was allowed among them. The Markov model was just able to achieve these transformations and to simulate the history of MS. Therefore, using a routine health-check longitudinal cohort, our study mainly focused on describing the natural history of MS, searching for the most likely component contributing to start the cascade confusions of MS, and predicting the effect of different initial states in 10 years by a reversible multi-state Markov model.

It is still controversy about which state the process of MS is most likely to begin with. A network-based study on a five-year follow-up longitudinal cohort identified central obesity and hypertension as the predominant MS risk factor cluster [22]. Lee-Ching Hwang et al. claimed that abdominal obesity or low HDL were the most likely factors to initiate the progress of MS in women [21]. Similarly, a Baltimore Longitudinal Study on Ageing demonstrated that abdominal obesity, low HDL cholesterol, or high triglycerides were the predictors of incidence of MS [28]. Limitations of these studies were that their subjects were confined to certain gender or age groups. Individuals in our research were divided into four age groups, 18–40 years, 40–49 years, 50–59 years, and ≥60 years, in two genders. We confirmed that the process of MS was most likely to begin with overweight or obesity and dyslipidemia in the young population (men <60 years and women <50 years). Also, we verified that the most likely beginning states of MS included hypertension state and 2-component state besides the above two states in the elderly population (men ≥60 years and women ≥50 years).

In the young people, obesity was defined as a main contributor in the process of MS, because it was suspected as a risk factor for hypertension, dyslipidemia and hyperglycemia [29]. Dyslipidemia could cause hypertension and hyperglycemia by mediating of free fatty acids, but the causality between dyslipidemia and obesity is still controversial. However, the beginning state changed with the growing age. The hormone level that affects individuals’ metabolism changes with age especially during menopause which results in the difference between the young and elderly. Chedraui Pet al. demonstrated that postmenopausal women with MS displayed higher IL-6 (inflammation) and lower urokinase-type plasminogen activator levels (endothelial dysfunction) which were mainly related to metabolic abnormalities and led to the occurrence of 2 components of MS at the same time [30]. Our research also found that men were more prone to develop MS than women of the same age while increasing age was an important factor inducing MS. The result was accordant with Scuteri Aet al. who studied 34,821 subjects from 12 cohorts in 10 European countries and one cohort from the USA in the MARE (Metabolic syndrome and Arteries RE search) Consortium [31].

We conducted a 10 years prediction with individuals beginning with different states of MS according to the existing transition probability. This is particularly important to prevent the occurrence of MS. In the predictions, individuals with the isolated hyperglycemia state had a greater chance to develop MS than the other isolated states, and almost reached the level of the individuals beginning with the 2-component state. Insulin resistance is the defined pathogenesis of diabetes and it commonly links the correlation to the other MS components. Several studies confirmed that four components of MS, obesity, hypertension, dyslipidemia, and hyperglycemia coexisted and influenced each other [32, 33]. There are some overlapping metabolic pathways in the pathogenesis of the four components of MS. Diabetes is often accompanied by hypertension and they share conjunct pathways such as the Renin-angiotensin-aldosterone System, Sympathetic Nervous System, adipokines, inflammatory pathway, and oxidative stress [34, 35]. A study conducted in the USA pointed out that hypertension occurs in approximately 50% to 80% of patients with type 2 diabetes and in 30% of patients with type 1 diabetes [36]. A search in Hong Kong was in line with it, as only 42% of people with diabetes could maintain normal blood pressure [37]. Hyperglycemia and dyslipidemia often occur in the same individual but its mechanism still remains controversial. Recently, it is suggested that cholesterol homeostasis plays an important role for beta-cells to perform adequate insulin secretion and low HDL cholesterol levels are normally related with hyperglycemia and type 2 diabetes [38]. Although there is no theory supporting that hyperglycemia could lead to obesity, central obesity had been confirmed to be the most relevant predisposing factor for insulin resistance [22, 39]. It should be particularly pointed out that glycemic control ought to attract more attention and that the individuals once diagnosed with hyperglycemia should receive active defense to prevent the emergence of the other components of MS.

We adopted CDS’ criteria to diagnose MS instead of international standard criteria in our study. Compared to Europeans and Americans, Chinese people have their own body shape characteristics. Therefore, CDS developed their own criteria which was also widely used in other studies [17, 18]. Previous study convinced that these two MS diagnostic criteria were in good accordance [24]. Simultaneous, considering the fact that we lack of waist circumference measurement data, CDS’ criteria could make our estimation more precise. Although CDS’ criteria we adopted may result slight difference in diagnoses of MS components compared to the international standard criteria. This difference would hardly effect on the overall developing trend of MS.

There are several limitations in our study. First, the person loss of follow-up was included in our study, and it may affect the interpretation of the transitional probability. We had used the averaging method to furthest reduce the impact of it. Secondly, the natural history of MS development may be not completely natural because of small part of interventions from the health services. Thirdly, mortality was not included in our model for the lack of the region specific information. Considering the general situation of mortality in our country, the influence is very small.

## Conclusions

The Markov model is able to give a good description of the evolutionary history of MS and to predict the future course based on the past evidence. Men are more prone to develop MS than women of the same age. The occurrence of the MS process mostly began with overweight or obesity and dyslipidemia in young people. In the elderly population, many individuals initiating with hypertension or 2 components besides the above two states. Individuals with the isolated hyperglycemia had a greater chance to develop MS than the other isolated MS’ components. These results indicate the importance of monitoring the component of MS.

## Electronic supplementary material

Additional file 1: Table S1: Basic characteristics of the study sample stratified by gender and age. **Table S2.** Annual transition probabilities (%) in Markov chain models for women in the 50–59 year group. **Table S3.** Annual transition probabilities (%) in Markov chain models for men in the ≥60 year group. **Table S4.** Annual transition probabilities (%) in Markov chain models for women in the ≥60 year group. (DOCX 21 KB)

Additional file 2: Figure S1: The predictive development of MS according to various starting components in men in the 40–49 year group. **Figure S2.** The predictive development of MS according to various starting components in women in the 40–49 year group. **Figure S3.** The predictive development of MS according to various starting components in men in the 50–59 year group. **Figure S4.** The predictive development of MS according to various starting components in women in the 50–59 year group. **Figure S5.** The predictive development of MS according to various starting components in men in the ≥60 year group. **Figure S6.** The predictive development of MS according to various starting components in women in the ≥60 year group. (DOCX 909 KB)
