# Supplementary material for: Description and prediction of the development of metabolic syndrome in Dongying City: a longitudinal analysis using the Markov model
Source: BMC Public Health. 2014 Oct 4;14:1033. doi: 10.1186/1471-2458-14-1033 (PMC4209018; doi:10.1186/1471-2458-14-1033)
Supplement: Supplementary file 1 — Additional file 1: Table S1: Basic characteristics of the study sample stratified by gender and age. Table S2. Annual transition probabilities (%) in Markov chain models for women in the 50–59 year group. Table S3. Annual transition probabilities (%) in Markov chain models for men in the ≥60 year group. Table S4. Annual transition probabilities (%) in Markov chain models for women in the ≥60 year group. (DOCX 21 KB) [file 12889_2014_7167_MOESM1_ESM.docx]

**Table S1.** Basic characteristics of the study sample stratified by gender and age

| Basic survey | <40 years | 40-49 years | 50-59 years | ≥60 years | F/x^2^ | *P*^b^ |
| --- | --- | --- | --- | --- | --- | --- |
| **Men** |  |  |  |  |  |  |
| N | 2438 | 1607 | 808 | 265 |  |  |
| Age(yeas) | 32.89±4.73 | 43.60±2.60 | 53.27±2.50 | 65.73±4.57 |  |  |
| Overweight or  Obesity | 1145 (46.96%) | 883 (51.84%) | 451 (55.82%) | 150 (56.60%) | 26.398 | <0.0001 |
| Hypertension | 585 (24.00%) | 659 (41.01%) | 428 (52.97%) | 165 (62.26%) | 350.763 | <0.0001 |
| Dyslipidemia | 719 (29.49%) | 629 (39.14%) | 291 (36.01%) | 83 (31.32%) | 43.259 | <0.0001 |
| Hyperglycemia | 91 (3.73%) | 200 (12.45%) | 147 (18.19%) | 49 (18.49%) | 206.176 | <0.0001 |
| MS in first year | 225 (9.23%) | 316 (19.66%) | 175 (21.66%) | 61 (23.02%) | 131.126 | <0.0001 |
| **Women** |  |  |  |  |  |  |
| N | 1216 | 786 | 216 | 174 |  |  |
| Age(yeas) | 33.02±4.74 | 43.54±2.53 | 53.84±3.01 | 65.34±4.40 |  |  |
| Overweight or  Obesity | 164 (13.49%) | 154 (19.59%) | 94 (43.52%) | 97 (55.75%) | 232.593 | <0.0001 |
| Hypertension | 84 (6.91%) | 123 (15.65%) | 89 (41.20%) | 104 (59.77%) | 409.258 | <0.0001 |
| Dyslipidemia | 77 (6.33%) | 76 (9.67%) | 61 (28.24%) | 72 (41.38%) | 237.949 | <0.0001 |
| Hyperglycemia | 18 (1.48%) | 25 (3.18%) | 38 (17.59%) | 46 (26.44%) | 261.858 | <0.0001 |
| MS in first year | 12 (0.99%) | 13 (1.65%) | 40 (18.52%) | 51 (29.31%) | 369.799 | <0.0001 |

^b^ P for each row testing the null hypothesis that values for six years were equal.

**Table S2.** Annual transition probabilities (%) in Markov chain models for women in the 50-59 year group

| Starting state | State after transition | | | | | | |
| --- | --- | --- | --- | --- | --- | --- | --- |
|  | No component | Isolated overweight or obesity | Isolated hypertension | Isolated dyslipidemia | Isolated hyperglycemia | 2 components | MS |
| No component | 69.18 | 5.41 | 5.95 | 9.73 | 0.54 | 7.57 | 1.62 |
| Isolated overweight or obesity | 19.61 | 41.18 | 1.96 | 3.92 | 1.96 | 19.61 | 11.76 |
| Isolated hypertension | 0 | 0 | 65.62 | 0 | 0 | 31.25 | 3.13 |
| Isolated dyslipidemia | 42.86 | 7.14 | 0 | 21.43 | 0 | 21.43 | 7.14 |
| Isolated hyperglycemia | 0 | 0 | 0 | 0 | 71.43 | 28.57 | 0 |
| 2 components | 0.56 | 3.39 | 20.90 | 1.69 | 0.56 | 53.13 | 19.77 |
| MS | 0 | 0 | 1.59 | 0 | 0.79 | 24.60 | 73.02 |

**Table S3.** Annual transition probabilities (%) in Markov chain models for men in the ≥ 60 year group

| Starting state | State after transition | | | | | | |
| --- | --- | --- | --- | --- | --- | --- | --- |
|  | No component | Isolated overweight or obesity | Isolated hypertension | Isolated dyslipidemia | Isolated hyperglycemia | 2 components | MS |
| No component | 65.98 | 6.19 | 11.34 | 5.15 | 2.06 | 6.19 | 3.09 |
| Isolated overweight or obesity | 20.63 | 49.21 | 0 | 1.59 | 0 | 26.98 | 1.59 |
| Isolated hypertension | 0 | 0 | 73.81 | 0 | 0 | 25.40 | 0.79 |
| Isolated dyslipidemia | 8.00 | 4.00 | 4.00 | 56.00 | 4.00 | 24.00 | 0 |
| Isolated hyperglycemia | 0 | 0 | 0 | 0 | 50.00 | 43.75 | 6.25 |
| 2 components | 1.58 | 2.77 | 11.07 | 0.79 | 1.98 | 55.72 | 26.09 |
| MS | 0 | 0 | 2.70 | 0 | 0.45 | 19.37 | 77.48 |

**Table S4.** Annual transition probabilities (%) in Markov chain models for women in the ≥60 year group

| Starting state | State after transition | | | | | | |
| --- | --- | --- | --- | --- | --- | --- | --- |
|  | No component | Isolated overweight or obesity | Isolated hypertension | Isolated dyslipidemia | Isolated hyperglycemia | 2 components | MS |
| No component | 58.34 | 8.33 | 8.33 | 13.33 | 1.67 | 8.33 | 1.67 |
| Isolated overweight or obesity | 25.81 | 51.60 | 3.23 | 0 | 0 | 16.13 | 3.23 |
| Isolated hypertension | 0 | 0 | 53.45 | 0 | 0 | 32.76 | 13.79 |
| Isolated dyslipidemia | 19.05 | 0 | 9.52 | 38.10 | 4.76 | 19.05 | 9.52 |
| Isolated hyperglycemia | 0 | 0 | 0 | 0 | 38.46 | 30.77 | 30.77 |
| 2 components | 1.09 | 0.54 | 10.87 | 0 | 2.72 | 56.52 | 28.26 |
| MS | 0 | 0 | 2.83 | 0 | 0.94 | 19.81 | 76.42 |
