# Supplementary material for: Description and prediction of the development of metabolic syndrome in Dongying City: a longitudinal analysis using the Markov model
Source: BMC Public Health. 2014 Oct 4;14:1033. doi: 10.1186/1471-2458-14-1033 (PMC4209018; doi:10.1186/1471-2458-14-1033)
Supplement: Supplementary file 2 — Additional file 2: Figure S1: The predictive development of MS according to various starting components in men in the 40–49 year group. Figure S2. The predictive development of MS according to various starting components in women in the 40–49 year group. Figure S3. The predictive development of MS according to various starting components in men in the 50–59 year group. Figure S4. The predictive development of MS according to various starting components in women in the 50–59 year group. Figure S5. The predictive development of MS according to various starting components in men in the ≥60 year group. Figure S6. The predictive development of MS according to various starting components in women in the ≥60 year group. (DOCX 909 KB) [file 12889_2014_7167_MOESM2_ESM.docx]

**
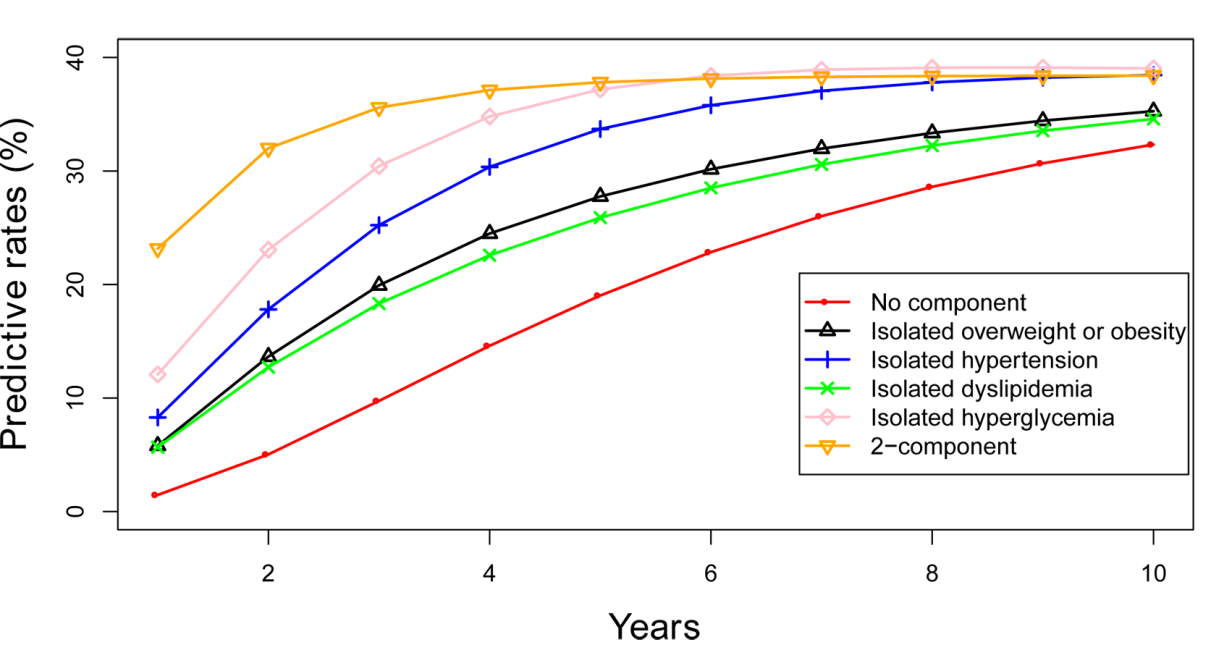
**

**Figure S1.** The predictive development of MS according to various starting components in men in the 40-49 year group


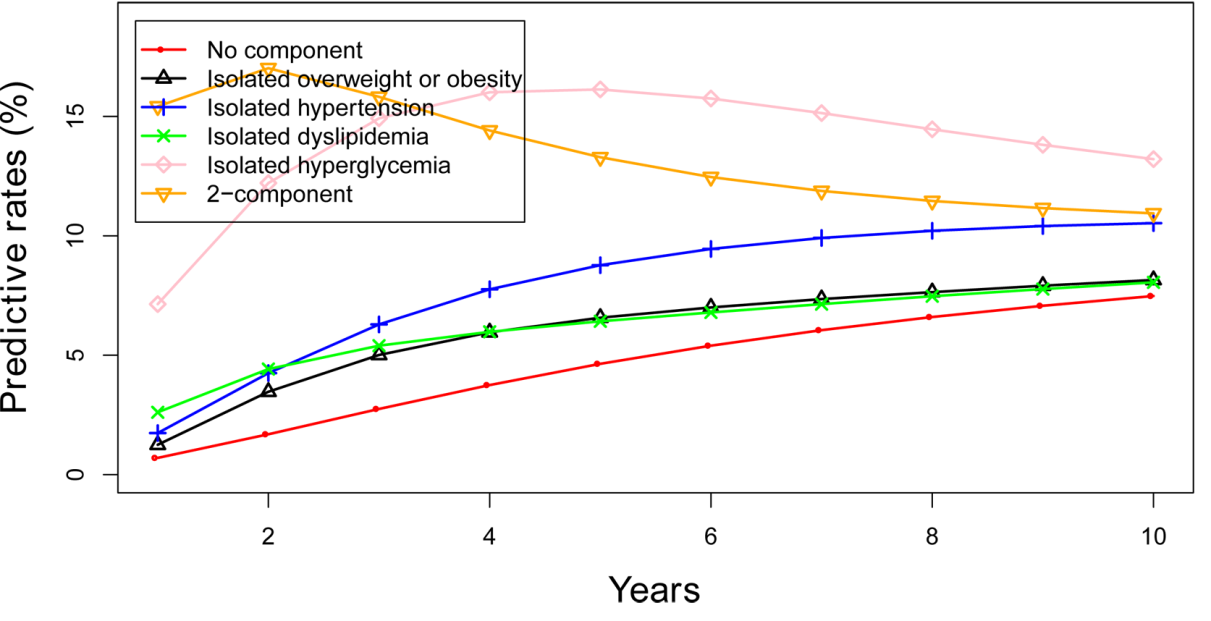


**Figure S2.** The predictive development of MS according to various starting components in women in the 40-49 year group


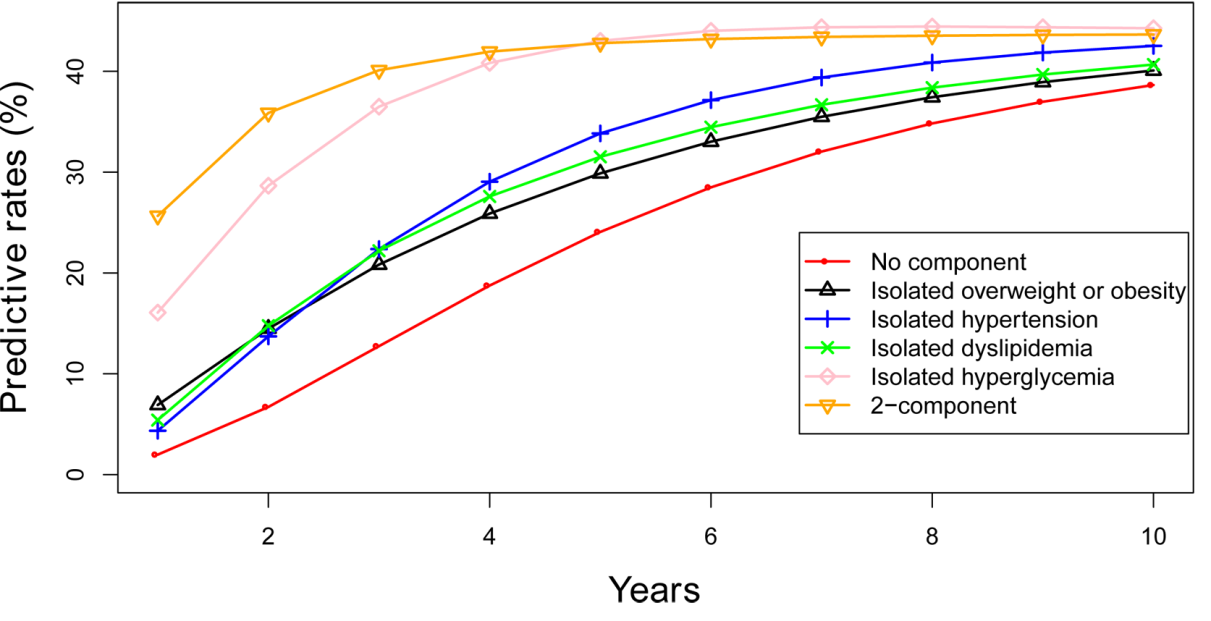


**Figure S3.** The predictive development of MS according to various starting components in men in the 50-59 year group


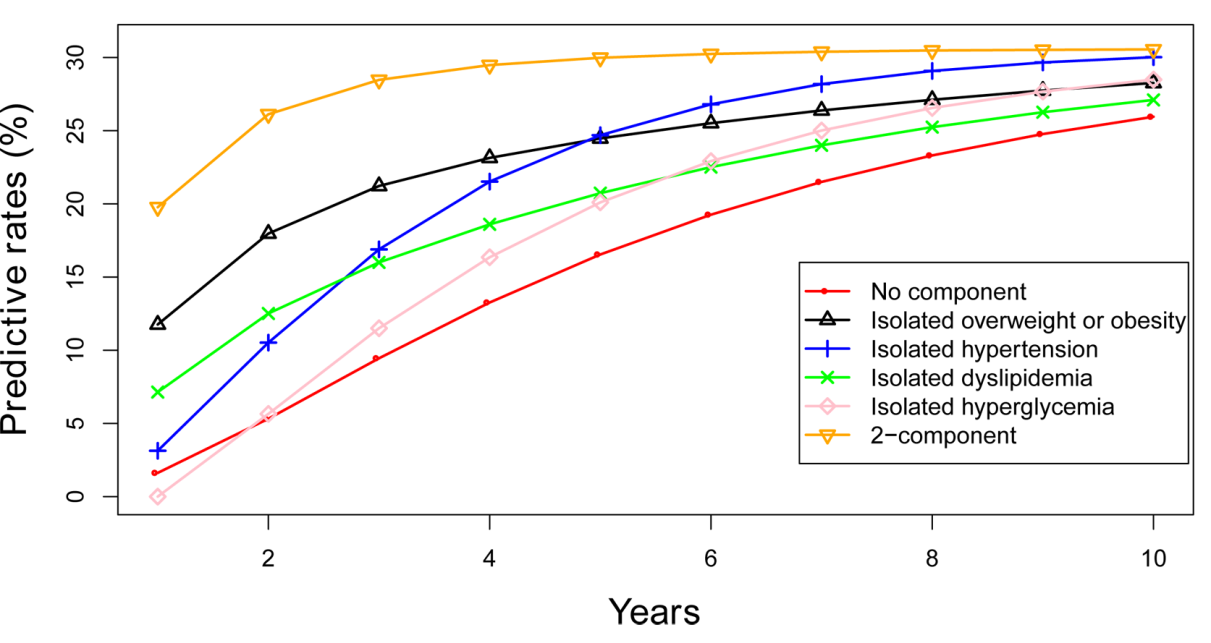


**Figure S4.**The predictive development of MS according to various starting components in women in the 50-59 year group


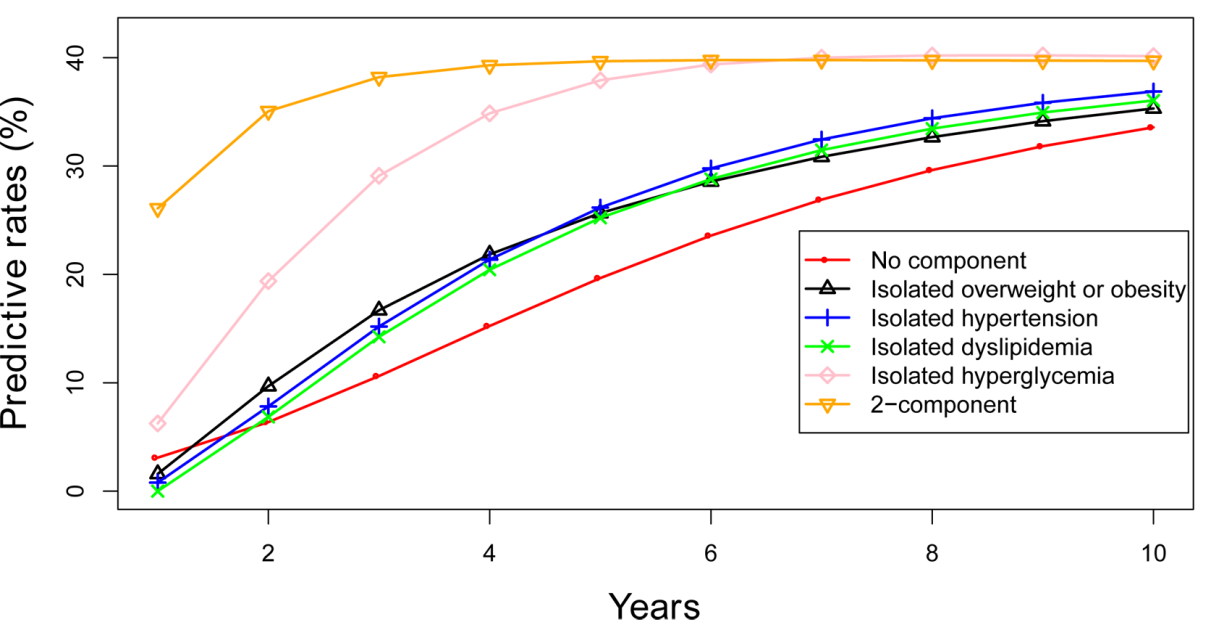


**Figure S5.** The predictive development of MS according to various starting components in men in the ≥60 year group


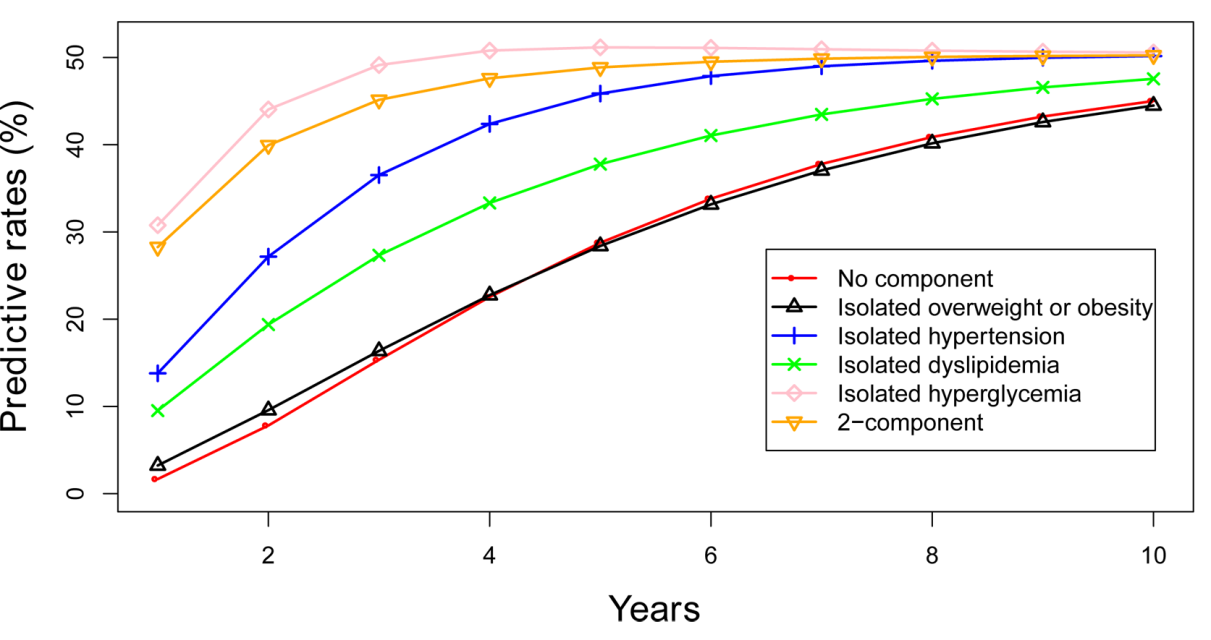


**Figure S6.** The predictive development of MS according to various starting components in women in the ≥60 year group
